# Conceptualizing the Value of Aging: What is it Like to Be An Older Woman in the 21st Century?

**DOI:** 10.1093/geroni/igab046.3474

**Published:** 2021-12-17

**Authors:** Karen Shilvock-Cinefro

**Affiliations:** George Williams College/Aurora University

## Abstract

The basis for this hermeneutic phenomenological research was to identify common themes in women 65 years of age and older and determine whether these women felt valued in ageing. The best description of this research becomes the study of the phenomena as real living or “entering the lifeworld “(Finlay, 2012). The research sought to increase the knowledge of how older women feel about their own ageing and the effect of society’s response to them. The participants covered a large age range of 66 to 93 years of age all of which experienced physical, emotional, and social changes involving age. The participants' response to these changes of ageing and society’s response to them ranged from very positive to very distressing. Twelve women were interviewed from a vast range of locations throughout the United States through zoom due to COVID 19. Their responses reflect four main categories: ageism, successful ageing, active engagement, and social support. The interviews focused on seven main questions: Tell me about being your age? Is there a time you can recall when you first felt older? Have you ever felt mistreated as an older adult? Have you ever felt you were discounted or ignored as being an older woman? Have you done anything to maintain your youth? Have you done anything to feel youthful? Have you ever felt dismissed related to your age? Utilizing seven questions and the conversation with these questions brought forth this study. This became a platform for these women to tell their stories.

